# New Insights in PRRT: Lessons From 2021

**DOI:** 10.3389/fendo.2022.861434

**Published:** 2022-04-05

**Authors:** Giulia Puliani, Alfonsina Chiefari, Marilda Mormando, Marta Bianchini, Rosa Lauretta, Marialuisa Appetecchia

**Affiliations:** ^1^ Oncological Endocrinology Unit, Scientific Institute for Research, Hospitalization and Healthcare (IRCCS) Regina Elena National Cancer Institute, Rome, Italy; ^2^ Department of Experimental Medicine, Sapienza University of Rome, Rome, Italy

**Keywords:** peptide receptor radionuclide therapy, radioligand therapy, predictive factors, prognostic factors, neuroendocrine tumors, neuroendocrine neoplasms, safety

## Abstract

Peptide receptor radionuclide therapy (PRRT) using radiolabeled somatostatin analogs has been used for over two decades for the treatment of well-differentiated neuroendocrine tumors (NETs), and the publication of the NETTER-1 trials has further strengthened its clinical use. However, many aspects of this treatment are still under discussion. The purpose of this review is to collect and discuss the new available evidence, published in 2021, on the use of ^177^Lu-Oxodotreotide (DOTATATE) or ^90^Y-Edotreotide (DOTATOC) in adult patients with NETs focusing on the following hot topics: 1) PRRT use in new clinical settings, broaden its indications; 2) the short- and long-term safety; and 3) the identification of prognostic and predictive factors. The review suggests a possible future increase of PRRT applications, using it in other NETs, as a neoadjuvant treatment, or for rechallenge. Regarding safety, available studies, even those with long follow-up, supported the low rates of adverse events, even though 1.8% of treated patients developed a second malignancy. Finally, there is a lack of prognostic and predictive factors for PRRT, with the exception of the crucial role of nuclear imaging for both patient selection and treatment response estimation.

## 1 Introduction

Peptide receptor radionuclide therapy (PRRT) with radiolabeled somatostatin analogs has been used for two decades for the treatment of well-differentiated neuroendocrine tumors (NETs) expressing somatostatin receptor (SSTR) type 2. The two most commonly used peptides are ^177^Lutetium (^177^Lu)-DOTATATE and ^90^Yttrium (^90^Y)-DOTATOC, respectively, beta- or gamma-emitting radionuclides (1). The publication of the NETTER-1 trial in 2017 has further confirmed the efficacy of this kind of therapy in NETs, also considering that the control arm of the study was represented by above label doses of somatostatin analogs, a very effective treatment (2). This study led to the approval of ^177^Lu-oxodotreotide (^®^Lutathera) by the European Medicines Agency and thus facilitated access to this treatment. However, this treatment is actually recommended only for progressive grade 1–2 gastroenteropancreatic (GEP) NETs. European Society for Medical Oncology (ESMO) guidelines recommend considering PRRT also in carcinoid syndrome (CS) and functional pancreatic (Pan) NETs refractory to somatostatin analogs and in selected cases of NET G3 (3).

Although PRRT represents a major cornerstone of treatment of well-differentiated low-grade GEP-NETs, some important aspects such as additional clinical indications, long-term safety, and predictive markers are not well established, and great attention is paid to these topics in recent literature.

This review aims to collect the evidence published in 2021 on the use of 177Lu-DOTATATE or 90Y-DOTATOC in adult patients with NETs in order to summarize the new evidence in 3 main research fields: 1) the use of PRRT in new settings, to broaden clinical indications; 2) short- and long-term safety assessment; and 3) the identification of prognostic and predictive factors.

## 2 Methods

We searched the PubMed database for articles in English on PRRT published in 2021. The search strategies used were “peptide receptor radionuclide therapy” and “radioligand therapy.” The latest search was carried out on November 18, 2021. We have selected all the articles concerning the use of PRRT in human patients affected by neuroendocrine neoplasms of any origin regarding one of three reported topics. Articles not on humans, not using ^177^Lu or ^90^Y compounds, or on children were excluded from this review. Excluding duplicate articles, from the original number of 531 articles, 453 were excluded after abstract screening and 33 after full-text evaluation. Ultimately, 45 studies were included, as reported in **Supplementary Figure S1**.

## 3 Results

### 3.1 New Indications and Settings for Peptide Receptor Radionuclide Therapy

#### 3.1.1 Expanding the Clinical Indication

The overexpression of SSTRs in NETs other than GEP has led PRRT to be used in these neoplasms even if not actually approved.

A retrospective study evaluated the long-term outcome of ^177^Lu-DOTATATE in patients with paragangliomas (PGLs), demonstrating a disease control rate (DCR) of 67%. At 40 months, the observed progression-free survival (PFS) rate was 63% (95% CI: 30–96) and the overall survival (OS) rate was 65% (95% CI: 32–97) (4). These data were confirmed in a prospective phase II clinical trial (5), in which an overall DCR of 80% was observed (95% CI: 68.9–91.9) after a mean of five cycles of PRRT. Patients treated with ^177^Lu-DOTATATE showed a better OS compared with those treated with ^90^Y-DOTATOC (143 vs. 92 months). No high-grade renal and hematological toxicities occurred in both studies. Regarding the risk of PRRT-induced acute catecholamine crisis, premedication combining alpha- and beta-adrenergic blocking agents was effective in preventing this complication in a series of 5 patients (6).

A retrospective multicenter study aimed at evaluating the role of ^68^Ga-DOTATATE PET/CT in metastatic medullary thyroid cancer (MTC) and for patients’ selection for PRRT. Twenty-one of 71 patients, with tumor expressing SSTR, were treated with PRRT, with ^177^Lu-DOTATATE or ^90^Y-DOTATOC or both (median number of treatment cycles, 3; range, 1–4). At baseline, 10 patients had radiological and 3 had biochemical progression. After a median follow-up of 12 months, 12 patients had radiological progression, 1 had biochemical progression, and 3 patients died. The median time to treatment failure (including radiological or biochemical progression or death) was 14 months (95% CI: 8–25) without difference in terms of age, type of radionuclide, calcitonin serum level, or gallium avidity (7).

Bronchopulmonary NETs expressing SSTR may also benefit from PRRT. A retrospective study evaluated the role of combined ^68^Ga-DOTATATE and ^18^F-FDG PET/CT imaging to guide the choice of PRRT treatment in patients with typical and atypical carcinoids (TC and AC). About half of the patients (46% TC, 53% AC) were unsuitable for PRRT. In 16 patients who were treated with PRRT, DCR at 3 months was 85% with an OS of 54.6 months (95% CI 44–70). Patients with all lesions ^68^Ga-DODATATE positive and ^18^FDG PET/CT negative were less likely to develop disease progression (8).

Finally, patients with functioning tumors can benefit from PRRT. A retrospective cohort study that included patients with refractory CS, without evidence of disease progression, demonstrated a reduction in bowel movement frequency of more than 30% with PRRT in 47% of patients, also with a benefit on flushing. Importantly, no carcinoid crisis occurred with the use of short-acting octreotide subcutaneously between cycles (9).

#### 3.1.2 Peptide Receptor Radionuclide Therapy in the Neoadjuvant Setting

Promising evidence is emerging on the use of PRRT as a neoadjuvant treatment. In an open-label retrospective study, enrolling patients with unresectable GEP-NET, ^177^Lu-DOTATATE resulted in a significant tumor shrinkage, allowing primary tumor resection in 26.3% of the patients. Baseline significant response predictors were primary duodenal tumor site, the size of the primary tumor (<5 cm), absence of regional lymph node involvement, the size (≤1.5 cm) and number (≤3) of liver metastases, and ^18^F-FDG uptake (SUV_max_ <5) in the primary tumor (10).

#### 3.1.3 Retreatment With Peptide Receptor Radionuclide Therapy

Several uncontrolled studies have evaluated the outcome and feasibility of retreatment after an initial response to the first single course of PRRT followed by later disease progression (rechallenge with PRRT).

A retrospective study on 40 patients with advanced GEP-NETs with progressive disease after the first PRRT course demonstrated that the second PRRT course determined partial remission in 5% of patients, stable disease in 52.5% of patients, and disease progression in 42.5% of patients. The median OS was 122.1 months and was significantly longer in patients without uptake at ^18^F-FDG-PET CT (145.50 vs. 95.06 months, respectively) (11).

In a large Danish retrospective study, progression after the first PRRT course was seen in 62% of patients. Thirty-two patients were submitted to a second series of PRRT, and progression was observed in 64% of patients. The median PFS was 19 (range, 10–32) months. Interestingly, this study also included 8 patients who underwent a third PRRT series, with a PFS of 12 (range, 8–15) months (12).

Two meta-analyses have been published on the rechallenge with PRRT (the term “salvage treatment” was used in some articles included in the meta-analyses). A meta-analysis on 13 studies involving 560 patients evaluated the efficacy and safety of PRRT retreatment in patients with GEP-NETs, with encouraging results. Median pooled PFS was 12.52 months (95% CI: 9.82–15.22), and pooled DCR was 71% (95% CI: 66–75). The safety profile for retreatment was comparable to the initial PRRT, with grade 3–4 adverse events occurring in 5% (95% CI: 2–8) of patients (13).

Similar results emerged from another meta-analysis, including 9 studies on 426 patients. After PRRT retreatment, pooled DCR was 76.9% (95% CI: 72.3–81.0) months, pooled PFS was 14.1 (95% CI: 12.2–15.9) months, and pooled median OS of 26.8 (95% CI: 18.8–34.9) months. As expected, PRRT showed a significantly lower DCR and shorter PFS compared to initial PRRT, without significant differences in hematologic and renal toxicities (14).

#### 3.1.4 Positioning Peptide Receptor Radionuclide Therapy in the Treatment Sequence

Until now, the optimal treatment sequence for NETs is not well established. A retrospective study in patients with metastatic G2 Pan-NETs, treated with more than one systemic therapy, showed that patients who received PRRT in the treatment sequence (most frequently as third or fourth line) had significantly prolonged survival compared with those who did not receive PRRT [median, 84 vs. 56 months; hazard ratio (HR), 0.55; 95% CI: 0.31–0.98] (15).

Parghane et al. (16) evaluated the long-term outcome of a combined chemotherapy and PRRT protocol with a “sandwich” regimen in the treatment of metastatic progressive NETs with both ^18^F-FDG and ^68^Ga-DODATOC avid lesions. In 38 patients analyzed, DCR of 84%, PFS of 72.5%, and OS of 80.4% at 36 months were observed. A longer PFS and higher DCR were noted in patients without metastatic bone involvement. Only low-grade and transient toxicities were registered, without renal toxicities of any grade (16).

The features of the main articles on the new indications and settings and safety of PRRT are summarized in **Table 1**.

**Table 1:** List of the main studies published in 2021 on **(A)** additional indications, **(B)** neoadjuvant role, **(C)** rechallenge, and **(D)** safety of PRRT treatment in patients affected by NETs.

Authors (ref)	Design	Patients^a^ total (M/F) number	Age Median (range) years	NET type	NET Grade	Prior treatment n (%)	PRRT Scheme Radionuclide, median dose, median n cycles	Main inclusion criteria	Aim of the study	Follow-up Median (range) months
***A) Additional indications of PRRT***										
Parghane RV (4)	R	10 (5/5)	49 (33–61)	Metastatic PGLs	–	RT: 6 (60%)	**^177^Lu:** 10 (100%)	Negative ^131^I-MIBG SPECT positive ^68^Ga-PET	PFS	40 (NA)
						CHT: 1 (10%)	24.42 GBq (range 7.4–37)		OS	
							in 4 (1–6) cycles			
Severi S (5)	P	46 (20/26)	52 (NA)	Progressive locally advanced or metastatic PGLs	–	NA	**^90^Y**: 12 (26%)	SSTR2 positive	Activity and safety	73 (5–146)
	(Ph 2)						9.2 GBq			
							in 5 cycles			
							**^177^Lu**: 34 (74%)			
							24.42 GBq			
							in 5 cycles			
Hayes AR (7)	R	21 (14/7)	50 (27–74)	MTC	–	NA	**^90^Y**: 5 (24%)	SSTR2 positive evaluated by ^68^Ga-PET	Role of ^68^Ga- PET in MTC	12 (2–47)
							**^177^Lu**: 12 (57%)			
							**Both**: 4 (19%)			
							in 3 cycles (1–4)			
Zidan L (8)	R	56 (24/32)	TC: 63(21–81)	Lung carcinoids	TC: 22 (39%)	Surgery ± SSA: 25 (44.6%)	**^177^Lu**: 14 (87.5%)	Progression or uncontrolled symptoms	Role of ^68^Ga- PET and ^18^F-FDG PET for treatment selection	TC: 37 (NA)
		(*only 16 treated by PRRT*)	AC: 68.5 (33–83)		AC: 34 (61%)	CHT: 3 (5.4%)	in 4 cycles (3–4)			AC: 38 (NA)
							**Both**: 2 (12.5%)			
Zandee WT (9)	R	22 (12/10)	62.7 ± 8.2^b^	Metastatic midgut NET with CS	G1: 7 (32%)	CHT: 2 (9%)	**^177^Lu:** 22 (100%)	Non-progressive and SSA refractory CS	Efficacy for symptoms reduction	>1 year
					G2: 7 (32%)	Other: 8 (37%)	27.8-29.6 GBq			
					UK: 8 (36%)		4 cycles			
***B) PRRT as neoadjuvant treatment***										
Parghane RV (10)	R	57 (33/24)	51.5 (30–78)	unresectable GEP-NET	G1: 26 (45.6%)	CHT: 15 (26%)	**^177^Lu**: 57 (100%)	Unresectable GEP NET with or without liver metastasis	Efficacy of neoadjuvant PRRT	24 (NA)
				P: 32 (56.1%)	G2: 30 (52.6%)	SSA: 12 (21%)	22.2-27.5 GBq			
				GI: 25 (43.9%)	G3: 1 (1.7%)		(14.8–40.7)			
							in 4 cycles (2–5)			
***C) Rechallenge with PRRT***										
Rodrigues M (11)	R	40 (26/14)	54.6 (29–83)	Advanced GEP	G1: 2 (5%)	LRT: 16 (40%)	**^177^Lu**: 40 (100%)	At least two courses of PRRT	Efficacy of a second PRRT	NA
				P: 18 (45%)	G2: 29 (72.5%)		cumulative 48.8 ± 11.8^b^ GBq			
				GI: 22 (55%)	G3: 8 (20%)					
					UK: 1 (2.5%)					
Zacho MD (12)	R	133 (72/61)	70 (64–76)	P: 31 (23.3%)	G1: 24 (20%)	SSA: 113 (85%)	***First series***	Patients treated at least one course PRRT	Treatment response	NA
				GI: 82 (61.6%)	G2: 78 (63%)	IFN: 42 (37%)	**^177^Lu:** 60 (45%)			
				Lung: 14 (10.5%)	G3: 21 (17%)	CHT: 67 (51%)	**^90^Y**: 66 (50%)			
				Oth: 6 (4.5%)		LRT: 11 (8%)	**Both**: 7 (5%)			
							***Second series***			
							**^177^Lu**: 25 (69%)			
							**^90^Y**: 8 (23%)			
							**Both**: 3 (8%)			
							***Third series***			
							**^177^Lu:** 6 (75%)			
							**^90^Y:** 2 (25%)			
***D) Safety of PRRT***										
Kovan B (17)	R	36 (18/18)	54.7 ± 12.9^b^	P: 8 (22.2%)	NA	NA	**^177^Lu**: 36 (100%)	NET patient treated with PRRT	Evaluating critical organ threshold values	20 (2–61)
				GI: 2 (5.5%)			691 ± 257^b^ mCi			
				MTC: 6 (16.7%)			in 3.91 ± 1.33^b^ cycles			
				Lung: 2 (5.5%)						
				UK: 18 (50%)						
Paganelli G (18)	P	43 (28/15)	65 (44–82)	GI NETs	G1: 13 (30%)		**^177^Lu**: 43 (100%)	Positive octreoscan or ^68^Ga- PET	DCR and Toxicity	118 (12.6–139.6)
	(Ph 2)				G2: 18 (42%)		27.5 GBq (25 pts)			
					UK: 12 (28%)		18.5 GBq (18 pts)			
							in 5 cycles			
Nilica B (19)	R	102 (67/35)	44 pts ≥65 years	GI: 47 (46.1%)	NA	NA	**^177^Lu**: 86 (84%)	≥4 PRRT cycles	Long-term safety	>52 weeks
				P: 24 (23.5%)			29.6 GBq in 4 cycles^d^	No concomitant oncologic treatment (excl. SSA)		
				Lung: 5 (4.9%)			**^90^Y**: 16 (16%)	≥52 weeks FU		
				PGLS: 3 (2.9%)			16 GBq in 4 cycles^d^			
				MTC: 1 (1%)						
				FTC: 3 (2.9%)						
				UK: 6 (5.9%)						
				NA: 13 (12.7%)						
Guhne F (20)	R	32 (16/16)	64.2 ± 11.1^b^	For: 16 (50%)	NA	NA	**^177^Lu**: 32 (100%)	Availability of ^68^Ga-PET after third cycle	Safety	NA
				Mid: 6 (18.7%)			20.7 ± 3.7 GBq			
				UK: 4 (12.5%)			in 3 cycles			
				Others: 6 (18.7%)						
Chantadisai M (21)	R	1631	59 (32-70)	GI: 11 (37%)	G1: 8 (27%)	SSA: 12 (40%)	**^90^Y:** 3 (10%)	Development of therapy-related hematologic neoplasms	OS	55 (17–145)
		[30 pts developed		P: 13 (43%)	G2: 10 (33%)	1-line CHT: 8 (27%)	10.5 GBq in			
		t-MN 15/15)]		Lung: 1 (3%)	NET G3: 1 (3%)	>1-line CHT: 3 (10%)	4 cycles			
				UK: 2 (7%)	UK: 11 (37%)	Others 11 (37%)	**^177^Lu**: 8 (27%) 22.1 GBq in 3.5 cycles			
				Oth: 3 (10%)		No: 5 (17%)	**Both**: 18 (60%) 25 Gbq in 5 cycles^c^			
Elston MS (22)	Cohort	34 PRRT (23/11)	65.1 (56.1–71.7)	GI: 13 (38.2%)	NA	CHT: 17 (50%)	NA	Unresectable NET without pituitary disease	Prevalence of hypopituitarism	68 (NA)
				P: 18 (52.9%)			31.8 (31.2–35.0) in 4 cycles (4–4.25)			
				Lung: 1 (2.9%)						
				UK: 2 (5.9%)						
		32 no PRRT (15/17)	61.6 (54.9–68.7)	GI: 18 (56.2%)	NA	CHT: 1 (3.1%)	–			
				P: 10 (31.2%)						
				Lung: 1 (3.1%)						
				UK: 3 (9.4%)						
Sundlov A (23)	P	68 (37/31)	66 (41–80)	GI: 40 (59%)	G1–G2	1-line CHT: 4 (6%)	**^177^Lu:** 68 (100%)	Progressive NET with SSRT expression	Evaluate long –term pituitary function after PRRT	30 (11–39)
	(Ph 2)			P: 14 (21%)		2 lines CHT: 2 (3%)	37 Gbq (14.8–66.6) in 5 cycles (2–9)			
				Lung: 5 (7%)		3 lines CHT: 2 (3%)				
				Oth: 9 (13%)		SSA: 55 (81%)				
						LRT: 27 (40%)				
						Others: 11 (16%)				
Jafari E (24)	R	13 (9/4)	52 (27–71)	NA	NA	NA	**^177^Lu**: 13 (100%)	NET treated by PRRT	Evaluating PRRT cardiotoxicity	21 (4–28)
							14.8 GBq (6–44) in 2 cycles (1–6)			
Fross-Baron K (25)	R	102 (64/38)	57.1 (29–79)	P: 102 (100%)	G1: 2 (1.9%)	1-line CHT: 68 (66.7%)	**^177^Lu:** 102 (100%)	Patients had previously received one (67%) or multiple (33%) chemotherapy lines prior to ^177^LuPRRT	PFS	34 (4–160)
					G2: 76 (74.5%)	2-lines CTH: 29 (28.4%)	32 ± 10.9 GBq, in 4 cycles (44 patients >4 cycles)		OS	
					G3: 7 (6.9%)	3-lines CHT: 5 (4.9%)				
					UK: 17 (16.7%)	Other: 16 (15.7%)				
						LRT: 39 (38.2%)				
						RT: 4 (3.9%)				
Chen L (26)	R	71 (42/29)	70 (55–80)	GI: 55 (77.5%)	G1 or TC: 38 (53.5%)	SSA: 66 (93%)	**^177^Lu**: 71 (100%)	>70 years	Safety	29 (NA)
				P: 8 (11.3%)	G2 or AC: 29 (40.8%)	CHT: 10 (14.1%)	29.6 GBq		QOL	
				Lung: 3 (4.2%)	G3: 2 (2.8%)	90Y: 3 (4.2%)	(78.9% of patients completed 4 cycles)		Efficacy	
				UK: 5 (7%)	UK: 5 (7%)					
Kipnis ST (27)	R	78 (39/39)	59.8 (53.5–69.2)	GI: 34 (43.6%)	G1: 27 (34.6%)	SSA: 49 (62.8%)	**^177^Lu:** 78 (100%)	Metastatic NETs with at least 1 dose of PRRT	PFS	15.5 (8.7–19.8)
				P: 22 (28.2%)	G2: 35 (44.9%)	LRT: 49 (62.8%)	29.6 GBq in 4 cycles^d^		OS	
				Oth: 22 (28.2%)	G3: 8 (10.3%)					
					UK: 8 (10.3%)					

R, retrospective; LRT, locoregional therapy; OS, overall survival; PFS, progression-free survival; PGL, paraganglioma; CS, carcinoid syndrome; SSA, somatostatin analogs; SI, small intestine; NA, not available in the article; UK, unknown; P, pancreas; CHT, chemotherapy; G, grade; GI, gastrointestinal; MTC, medullary thyroid cancer; LRT, locoregional treatment; Pts, patients; FU, follow-up; FTC, follicular thyroid cancer; for, forgut; mid, midgut; t-MN, therapy-related myeloid neoplasm; RT, radiotherapy; Oth, other; DCR, disease control rate. ref, reference; M, males; F, females; NET, neuroendocrine tumor; ph, phase; PRRT, peptide receptor radioligand therapy; n, number; QOL, quality of life; PET, positron emission tomography; SSTR, somatostatin analogs receptor.

a referred to patients treated by PRRT, unless otherwise stated; ^b^mean ± standard deviation; ^c^1 patient received 36.5 GBq; ^d^reported protocol.

### 3.2 Short- and Long-Term Safety of Peptide Receptor Radionuclide Therapy

The critical organs to consider before PRRT are the kidney and bone marrow. Until now, the accepted upper limit doses have been adapted by external radiotherapy (23 Gy for kidneys and 2 Gy for bone marrow) (28). Data from a retrospective study including 37 patients receiving ^177^Lu-DOTATATE showed that only 5.5% reached 2 Gy to the bone marrow and the threshold value of 23 Gy for kidney was reached in 21% of patients receiving 4 cycles and in 37.5% in case of more than 4 cycles. However, no long-term renal dysfunction occurred with a kidney dose of 23–29 Gy, suggesting a possible increase of kidney threshold levels (17). Accordingly, an open-label, prospective, phase II study showed the absence of grade 3–4 hematological toxicities and renal impairment using ^177^Lu-DOTATATE at two different doses (18.5 and 27.5 GBq in 5 cycles) (18). Globally, PRRT was safe, with a low incidence of severe nephrotoxicity and hematotoxicity. Notably, in the majority of the studies, a protocol of amino acid infusion was used in order to reduce renal injury. A large study described an impairment in kidney function and hemoglobin in 20.6% of patients 1 year after the start of the treatment. Age over 65 years seems to be a risk factor for the development of anemia. Leukocyte and platelet count reduction was 14.7% and 10.8% of patients, respectively (19). Another retrospective study did not confirm any significant change in glomerular filtrate after PRRT (20). Considering the late effects of PRRT, in a large series of 1,631 treated patients, only 1.8% developed therapy-related myeloid neoplasm, including myelodysplastic syndrome and acute myeloid leukemia, after a median time of 43 months (range, 6–123) (21). A case series on 5 patients with bone marrow infiltration of NETs and myelosuppression demonstrated that PRRT could be safe also in these patients when prophylactic peripheral blood stem cell collection was performed before PRRT (29). In another study, grade 1–2 hematological toxicities were observed in 60.3% of patients and grade 3–4 toxicities were observed in 25 patients (32.1%), without the development of myelodysplasia or the need for dialysis or liver failure (27).

Two studies have evaluated the effect of PRRT on pituitary function, as normal pituitary tissue expresses SSTRs. Comparing patients treated or not with PRRT, after a long follow-up (68 months), the prevalence of hypopituitarism was the same in the two groups (22). Another study evaluated pituitary function at baseline and 1 year after high-dose PRRT. The study demonstrated a significant decrease in insulin like growth factor 1 (IGF1) levels, which was related to the number of cycles and the absorbed radiation dose, without changes in the adrenal and thyroid axes (23).

Strosberg et al. (30) reported a 3% incidence of risk of bowel obstruction within 3 months in patients receiving PRRT. All patients had a mesenteric or peritoneal disease and responded to high doses of corticosteroid (30). PRRT-related cardiotoxicity has been investigated in 13 patients affected by NETs. No significant change in serum troponin I was demonstrated after PRRT (24).

The safety of ^177^Lu-DOTATATE was also confirmed in patients with advanced PanNET heavily pretreated with chemotherapy. Grade 3–4 bone marrow toxicities occurred in 10.8% and were unrelated to the type and duration of previous chemotherapy, amount of activity administered, and dose absorbed from the bone marrow. One patient (1.0%) developed acute myeloid leukemia (25). In older patients (≥70 years) treated with PRRT, the most common adverse events were fatigue and grade 1–2 gastrointestinal disturbances, occurring in 98.3% of patients. The most common hematological adverse events were grade 1–2 lymphocytopenia and anemia. An increase in creatinine values after PRRT occurred in 12.7% of patients (grade 1–2) (26). In a small study evaluating the combination of ^177^Lu-DOTATATE and ^90^Y-DOTATOC therapy in 9 patients affected by NETs with a large bulky lesion (≥5 cm), posttreatment imaging showed excellent uptake of the radionuclides in the lesions in almost all patients, and only mild-grade adverse events were observed (31).

The frequencies of adverse events described in the main studies are summarized in **Supplementary Table S1**.

### 3.3 Prognostic and Predictive Factors

Many studies have focused on the role of factors that could predict prognosis or response to PRRT, including circulating biomarkers, clinical parameters, and imaging.

Starting from the role of inflammation in NET progression, Ohlendorf et al. (32), in a study on 33 patients with advanced GEP-NETs treated with PRRT, evaluated the predictive role of inflammatory markers. C-reactive protein (CRP), composite index as Platelet × CRP multiplier (PCM), CPR/albumin ratio, and absolute neutrophil count were all significantly higher in patients who were non-responders to PRRT. Interestingly, in this study, the first ^68^Ga-DOTATATE PET/CT was performed early (after two cycles of treatment); at this time point, CRP and neutrophil-to-lymphocyte ratio were predictors of change in tumor burden (32). Another inflammatory biomarker, platelet-to-lymphocyte ratio (PLR), was evaluated in a retrospective study on a heterogeneous population of 42 patients affected by NET (all grades and sites) and treated by ^177^Lu-DOTATATE. Patients with PLR greater than 173.1 had significantly reduced PFS, with a univariate HR for progression or death of 3.82 (95% CI: 1.21–12.03) (33).

The predictive role of the classical neuroendocrine markers is debated. A study by Papantoniou et al. (34) demonstrated that changes in chromogranin A and 5-hydroxyindoleacetic acid during treatment were not predictors of PRRT response (34), although baseline values correlated to PFS (34, 35).

In the field of biochemical markers, growing attention is paid to NETest, an application of liquid biopsy in the field of NET, which has also demonstrated a prognostic role (36). In a larger study on the personalized approach to patients affected by neuroendocrine neoplasms, Frilling et al. (37) described that NETest scores decreased after 6 months in 9/9 patients with metastatic small bowel NETs treated with a combination of surgery and PRRT, and NETest values directly correlated with tumor volume. On the contrary, cell-free DNA levels, although higher in patients with NETs than those in healthy controls, were unable to predict OS and response to PRRT (38).

Many clinical parameters have been proposed as prognostic markers. Factors associated with a reduction in PFS and OS in PRRT-treated patients were ascites (35), marked liver metastasis burden (18, 25, 35), unusual metastatic sites (35), and age >65 years at the time of PRRT (18). Other factors such as interim ascites, the presence of ≥5 bone metastases, and NETs other than GEP were predictors of worse OS (35). The importance of bone metastasis is also confirmed by the evidence that an increase in baseline alkaline phosphatase is associated with poorer PFS and OS (25, 26). Das et al. (39) developed an interesting clinical score that included 5 elements, availability of treatments other than ^177^Lu-DOTATATE, prior systemic treatments, symptoms, tumor burden of critical organs, and peritoneal carcinomatosis, that was able to predict PFS only in patients treated with PRRT. One study failed in demonstrating the role of sarcopenia and myosteatosis in predicting PFS in 49 patients with NET (any grade) treated by PRRT (40). Finally, one study confirmed that resection of the primary tumor had a beneficial effect in increasing OS after PRRT (41).

Morphological and functional imaging has been proposed for treatment response prediction. In a study on 66 patients with PanNET undergoing PRRT, the authors evaluated the tumor growth rate (TGR), expressed as change/month. TGR decreases significantly during PRRT with ^177^Lu-DOTATATE, and patients with TGR ≥0.5%/month had shorter PFS (HR, 2.82; 95% CI: 1.05–7.57) (42). Many studies focused on the prognostic role of ^18^F-FDG PET/CT status even in the setting of PRRT-treated patients (18, 43, 44). An interesting prospective 10-year follow-up study of 166 patients demonstrated that ^18^F-FDG PET/CT is more effective than grading in predicting OS and PFS. In the subgroup of 78 patients who received PRRT, ^18^F-FDG PET/CT negative cases had significantly longer survival. Interestingly, PRRT increased OS in patients with positive ^18^F-FDG PET/CT when compared with non-treated patients, while no difference in OS was found between treated and not-treated subgroup of patients with negative ^18^F-FDG PET/CT (43). A recent meta-analysis on 12 studies and 1,492 patients evaluated the prognostic role of pretreatment ^18^F-FDG PET/CT in patients affected by any grade NETs treated with PRRT. Positive uptake at ^18^F-FDG PET/CT was associated with a higher risk of worse outcome [odds ratio (OR), 4.85; 95% CI: 2.27–10.36]. Regarding PFS, the pooled HR for progression was higher in case of positive ^18^F-FDG PET/CT (HR, 2.45; 95% CI: 1.48–4.04), and likewise, OS was lower (HR, 2.25; 95% CI: 1.55–3.28) (44). SSTR2 expression assessment by nuclear imaging is mandatory for selecting patients for PRRT. However, its prognostic value is less clear. Two studies evaluated the role of standardized uptake value (SUV) parameters at ^68^Ga-DOTATATE PET/CT in predicting PFS and response to treatment (45, 46). The mean SUV_max_ was significantly higher in responders than that in non-responders (45, 46) and was higher in patients with PFS >18 months (46). In a subset of 36 patients, another ^68^Ga-DOTATATE PET/CT scan was performed before the second cycle of PRRT, and SUV_max_ correlated to therapy response (45). Accordingly, another study demonstrated that the evaluation after two cycles can predict further response. With stable disease after 2 cycles, patients with PanNET were more likely than patients with other NETs to achieve a response (0.60 vs. 0.11) after 4 cycles. In patients with a response after two cycles, all PanNETs demonstrated a continuous response after 4 cycles compared with only 66% of other NETs (47).

PRRT absorbed dose may play a role in predicting the response. Both for small intestine and PanNETs, a dose–response relationship was found between the absorbed dose and tumor shrinkage, which was more pronounced in PanNET (48). Histological parameters have also been proposed as predictors of treatment response. The expression of SSTR2, assessed by immunohistochemistry in tumor samples, was not a predictive factor for PRRT response in a study on 42 patients with small intestine NETs (49). It has also been proposed that in unresponsive patients, PRRT may result in a clonal selection of resistant cells. In a case series on 7 patients with metastatic PanNET treated by PRRT and with evidence of progressive disease within 6 months from treatment, 3 patients underwent a new biopsy. In 2 cases, Ki 67 labeling index increased significantly, and in one patient morphology changed to poorly differentiated. The hypothesis of initial tumor heterogeneity was also supported by the positivity of both gallium and ^18^F-FDG PET/CT (50).

## 4 Conclusions

**Figure 1:**
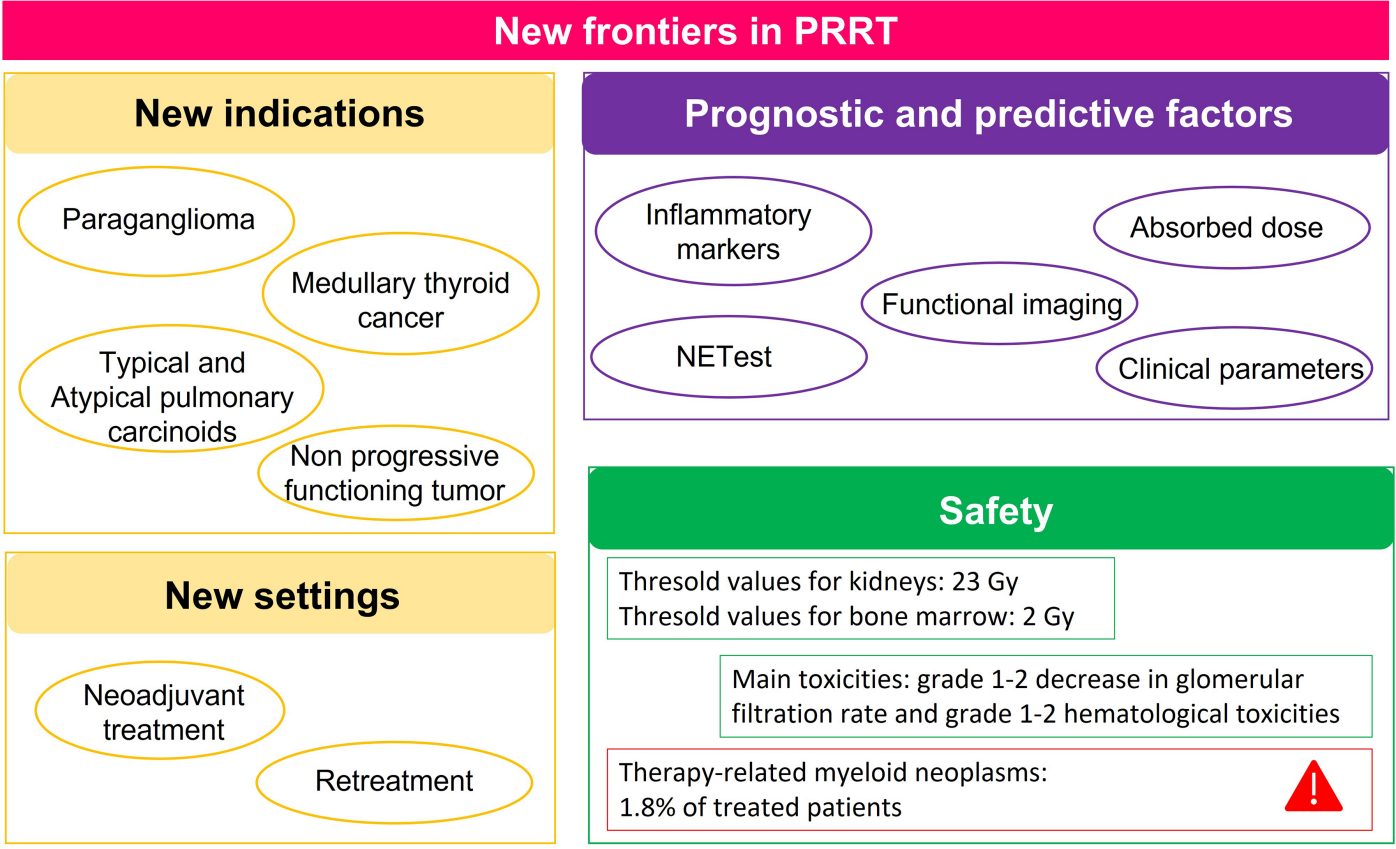
Summary of the main findings of articles published in 2021 on the 3 hot topics of peptide receptor radionuclide therapy (PRRT) in neuroendocrine tumors (NETs): new indications and settings, prognostic and predictive factors, and safety.

In 2021, many articles have been published on three hot topics of PRRT treatment in NETs: new clinical indications, safety, and prognostic and predictive markers. The main findings are summarized in **Figure 1**. Considering the evidence that this treatment has been used in PGLs, MTC, pulmonary carcinoids, and uncontrolled CS and in the neoadjuvant or salvage settings, PRRT indications are likely to increase in the near future. Despite the concern of the kidney and bone marrow toxicities of PRRT, available studies, including long follow-up studies, demonstrated the safety of this treatment, with the worse complication, the development of second neoplasia, appearing in 1.8% of treated patients. Finally, as in other aspects of NETs, prognostic and predictive factors are also lacking for PRRT. New evidence confirmed the crucial role of nuclear imaging not only for the selection of the patients but also for estimating treatment response.

## Author Contributions

Conceptualization: GP and MA. Data curation: GP, AC, MM, MB, and RL. Methodology and validation: GP. Writing—original draft: GP, AC, MM, MB, and RL. Writing—review and editing and supervision: MA. All authors contributed to the article and approved the submitted version.

## Conflict of Interest

MA does consultancy and has received research grants from Bayer, Eisai, and Eli-Lilly.

The remaining authors declare that the research was conducted in the absence of any commercial or financial relationships that could be construed as a potential conflict of interest.

## Publisher’s Note

All claims expressed in this article are solely those of the authors and do not necessarily represent those of their affiliated organizations, or those of the publisher, the editors and the reviewers. Any product that may be evaluated in this article, or claim that may be made by its manufacturer, is not guaranteed or endorsed by the publisher.

## Supplementary Material

Supplementary Table 1 Frequency of adverse events of PRRT reported in main studies.

Supplementary Figure 1 PRISMA flow diagram of the search strategy.
