# Supplementary material for: New Insights in PRRT: Lessons From 2021
Source: Front Endocrinol (Lausanne). 2022 Apr 5;13:861434. doi: 10.3389/fendo.2022.861434 (PMC9016202; doi:10.3389/fendo.2022.861434)
Supplement: Supplementary Table 1 — Frequency of adverse events of PRRT reported in main studies. [file Table_1.docx]

Supplementary Material

# Supplementary Table 1. Frequency of adverse events of PRRT reported in main studies.

|  | **Side effects** | **Frequency**  cases/total (%) | **Description** | **Reference** |
| --- | --- | --- | --- | --- |
| Nephrotoxicity | GFR decreased | 21/102 (20.6%) | Decrease in CDK grading | Nilica(1) |
|  |  | 9/71 (12.7%) | Grade 1 | Chen(2) |
|  |  | 0/102** | - | Fross-Baron(3) |
|  |  | 12/78 (15.4%) | Grade 2: 7; grade 3-4: 5^§^ | Kipnis(4) |
| Hematotoxicity | WBC decreased | 15/102 (14.7%) | Grade 1-2 | Nilica(1) |
|  |  | 14/71 (19.7%)* | Grade 1-2 | Chen(2) |
|  |  | 5/102 (4.9%)** | Grade 3 | Fross-Baron(3) |
|  |  | 26/78 (33.3%) | grade 2: 16; grade 3-4: 10^§^ | Kipnis(4) |
|  | Plt decreased | 11/102 (10.8%) | Grade 1 | Nilica(1) |
|  |  | 15/71 (21.1%) | Grade 1-2 (only 1 grade 3-4) | Chen(2) |
|  |  | 6/102 (5.9%)** | Grade 3: 5; grade 4:1 | Fross-Baron(3) |
|  |  | 9/78 (11.5%) | Grade 2: 2; Grade 3-4: 7^§^ | Kipnis(4) |
|  | Anemia | 21/102 (20.6%) | Grade 1-2 | Nilica(1) |
|  |  | 27/71 (38%) | Grade 1-2 | Chen(2) |
|  |  | 2/102 (1.9%)** | Grade 3 | Fross-Baron(3) |
|  |  | 16/78 (20.5) | Grade 2: 6; Grade 3-4: 10^§^ | Kipnis(4) |
| Liver Injury | AST/ALT | 9/71 (12.7%) | Grade 1-2 | Chen(2) |
|  | ALP/GGT | 19/71 (26.8%) | Grade 1-2 (only 1 grade 3-4) | Chen(2) |
|  | Hepatic failure | 1/102 (1%) | Grade 5 | Fross-Baron(3) |
|  | Biochemical liver injury | 6/78 (7.7%) | Grade 2^+§^ | Kipnis(4) |
|  |  | 6/78 (7.7%) | Grado 3-4^++§^ | Kipnis(4) |
| Fatigue | | 59/71 (83.1%) | Grade 1-2 | Chen(2) |
| Gastro-intestinal | nausea | 29/71 (40.8%) | Grade 1-2 | Chen(2) |
|  | vomiting | 4/71 (5.6%) | Grade 1-2 | Chen(2) |
|  | diarrhea | 27/71 (28%) | Grade 1-2 (only 2 grade 3-4) | Chen(2) |
|  | anorexia | 4/71 (5.6%) | Grade 1-2 | Chen(2) |
| Long-term side effect | Second malignancies | 30/1631 | AML: 7 (23%)  MDS: 23 (77%) | Chantadisai (5) |
|  |  | 0/78 (0%) | - | Kipnis(4) |
|  |  | 1/102 (1%) | AML | Fross-Baron(3) |

GFR**:** Glomerular filtration rate. Plt: platelet; WBC: white blood cells. Grade defines according to CTCAE, MDS: myelodysplastic syndrome; AML: acute myeloid leukemia; CKD: chronic kidney disease.

*: considering only neutropaenia: 7 (9.9%), grade 1-2, only 1 with grade 3-4; only lymohopaenia 41 (57.7%), of which 10 grade 3-4.

** Fross-baron reported only grade 3-4 toxicities.

^§^ Kipnis: evaluated only grade 2-4.

+ Biochemical liver injury was defined as the new development of a total bilirubin greater than 1.5x the upper limit of normal, AST greater than 3x the upper limit of normal, or ALT greater than 3x the upper limit of normal.

++Grade 3/4 biochemical liver injury was defined as the new development of a total bilirubin greater than 3x the upper limit of normal, AST greater than 5x the upper limit of normal, or ALT greater than 5x the upper limit of normal.

**References**

1. Nilica B, Svirydenka A, Fritz J, Bayerschmidt S, Kroiss AS, Gruber L, et al. Nephrotoxicity and Hematotoxicity One Year after Four Cycles of Peptide Receptor Radionuclide Therapy (Prrt) and Its Impact on Future Treatment Planning. A Retrospective Analysis. *Rev Esp Med Nucl Imagen Mol (Engl Ed)* (2021). doi: 10.1016/j.remn.2021.03.004.

2. Chen L, Navalkissoor S, Quigley AM, Gnanasegaran G, Mandair D, Toumpanakis C, et al. (177)Lu-Dotatate in Older Patients with Metastatic Neuroendocrine Tumours: Safety, Efficacy and Health-Related Quality of Life. *Eur J Nucl Med Mol Imaging* (2021) 48(11):3582-94. doi: 10.1007/s00259-021-05332-0.

3. Fross-Baron K, Garske-Roman U, Welin S, Granberg D, Eriksson B, Khan T, et al. 177lu-Dotatate Therapy of Advanced Pancreatic Neuroendocrine Tumors Heavily Pretreated with Chemotherapy: Analysis of Outcome, Safety, and Their Determinants. *Neuroendocrinology* (2021) 111(4):330-43. doi: 10.1159/000506746.

4. Kipnis ST, Hung M, Kumar S, Heckert JM, Lee H, Bennett B, et al. Laboratory, Clinical, and Survival Outcomes Associated with Peptide Receptor Radionuclide Therapy in Patients with Gastroenteropancreatic Neuroendocrine Tumors. *JAMA Netw Open* (2021) 4(3):e212274. doi: 10.1001/jamanetworkopen.2021.2274.

5. Chantadisai M, Kulkarni HR, Baum RP. Therapy-Related Myeloid Neoplasm after Peptide Receptor Radionuclide Therapy (Prrt) in 1631 Patients from Our 20 Years of Experiences: Prognostic Parameters and Overall Survival. *Eur J Nucl Med Mol Imaging* (2021) 48(5):1390-8. doi: 10.1007/s00259-020-05127-9.
