# Supplementary figures and images for: New Insights in PRRT: Lessons From 2021
Source: Front Endocrinol (Lausanne). 2022 Apr 5;13:861434. doi: 10.3389/fendo.2022.861434 (PMC9016202; doi:10.3389/fendo.2022.861434)

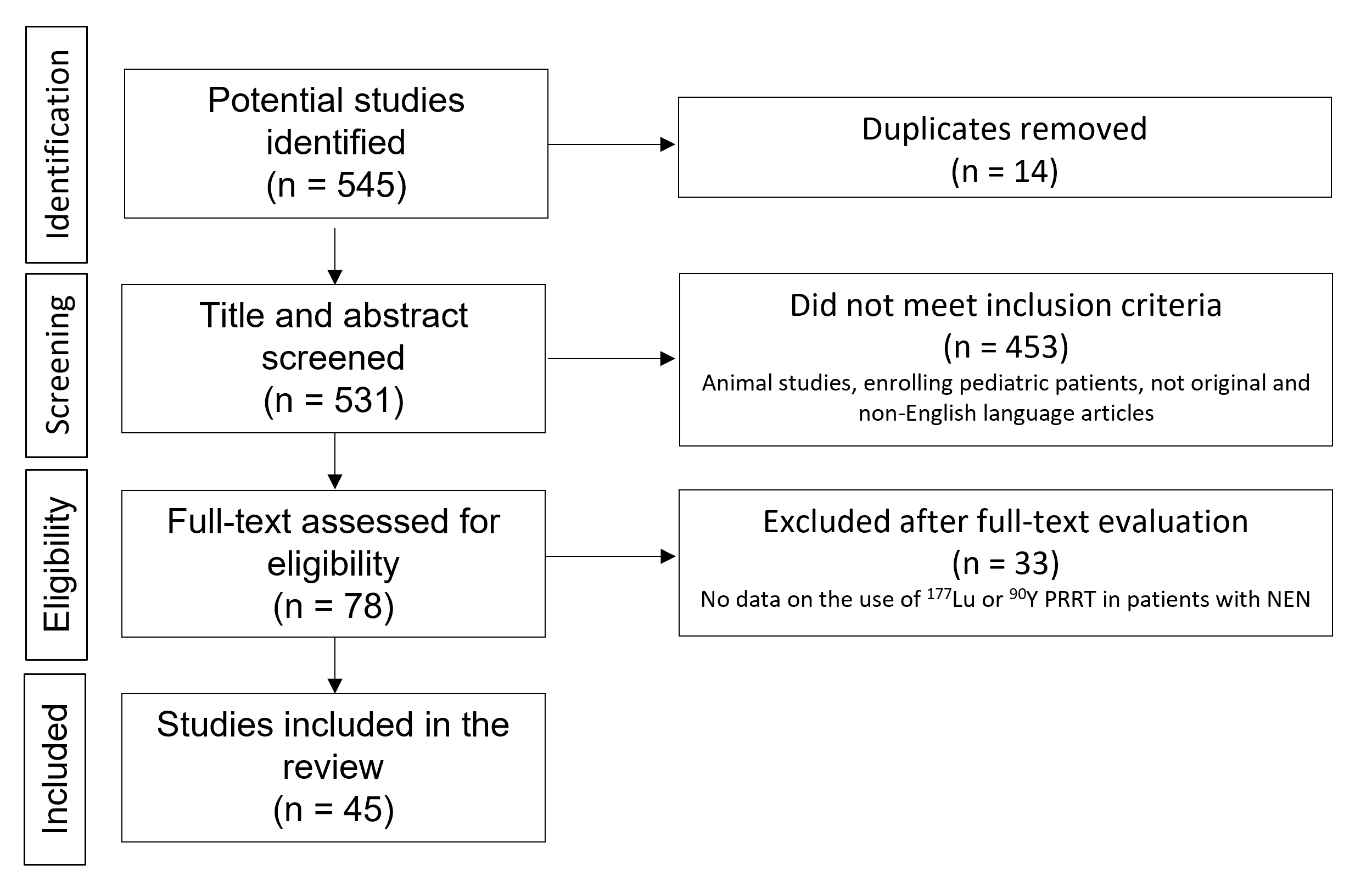

Supplement: Supplementary Figure 1 — PRISMA flow diagram of the search strategy. [file Image_1.tif]
